# Molecular simulations of peptides and proteins with Molecular Fragment Dynamics (MFD)

**DOI:** 10.1186/1758-2946-5-S1-P4

**Published:** 2013-03-22

**Authors:** Andreas Truszkowski, Annamaria Fiethen, Hubert Kuhn, Achim Zielesny, Matthias Epple

**Affiliations:** 1 Inorganic Chemistry and Center for Nanointegration, University of Duisburg-Essen, Essen, 45141, Germany; 2 CAM-D Technologies, Essen, 45117, Germany; 3 Institute for Bioinformatics and Chemoinformatics, Westphalian University of Applied Sciences, Recklinghausen, 45665, Germany

## 

Molecular Fragment Dynamics (MFD) is a mesoscopic simulation technique based on dissipative particle dynamics. The method has been shown to successfully describe the phase behaviour and interfacial tensions of large chemical systems in good agreement with experimental results [1]. Unlike molecular mechanics a MFD simulation is based on "coarse-grained" molecular fragments rather than "fine-grained" atom types.

The current MFD fragment definition is based on a SMILES like one-dimensional line notation which is visualized with the Structure Diagram Generator of the Chemistry Development Kit (CDK) [2]. For an extension of the MFD technique to chemical ensembles containing peptides and proteins an appropriate molecular fragment cheminformatics tool for their design is in need.

This work provides an editor for the construction of peptides and proteins from molecular fragments. It includes building blocks for all 20 proteinogenic amino acids, their charged species and disulfide bonds. A flexible input of one-letter and three-letter amino acid codes is supported. Additionally, the editor has the capability to set charges of side chains manually or automatically.

## References

[B1] Schulz SG The Phase Behavior of Amphiphilic Surfactants and Polymers: A Dissipative Particles Dynamics Study 2004 Dissertation, Essen, University of Duisburg-Essen

[B2] Steinbeck C Han Y Kuhn S Horlacher O Luttmann E Willighagen E The Chemistry Development Kit (CDK): An open-source Java library for chemo- and bioinformatics J Chem Inf Comput Sci 2003 43 493 500 10.1021/ci025584y 12653513 PMC4901983

